# Mechanistic insights into HPV-positivity in non-smokers and HPV-negativity in smokers with head and neck cancer

**DOI:** 10.3389/fonc.2024.1484319

**Published:** 2025-01-09

**Authors:** Markus Hoffmann, Susanne Hille, Asita Fazel, Martin Laudien, Susanne Wiegand, Martin Müller, Oliver J. Müller, Elgar Susanne Quabius

**Affiliations:** ^1^ Department of Otorhinolaryngology, Head and Neck Surgery, University Hospital Schleswig-Holstein, Kiel, Germany; ^2^ Quincke-Forschungszentrum (QFZ), Christian-Albrechts-University, Kiel, Germany; ^3^ Department of Internal Medicine IV, University Hospital Schleswig-Holstein, Kiel, Germany; ^4^ German Center for Cardiovascular Research (DZHK), partner site Kiel, University Hospital Schleswig-Holstein, Kiel, Germany; ^5^ Research Program Infection and Cancer, German Cancer Research Center, Heidelberg, Germany

**Keywords:** HPV, virus infection, viral cell entry, smoking, SLPI, Annexin A2, HNSCC

## Abstract

**Introduction:**

Several aspects of the involvement of HPV in the pathogenesis of HPV-associated diseases remain poorly understood including mechanistic aspects of infection and the question of why the majority of HPV-positive HNSCC-patients are non-smokers, whereas HPV-negatives are smokers. Our previous research, based on 1,100 patient samples, hypothesized an explanation for this phenomenon: Smoking induces upregulation of a mucosal protective protein (SLPI), which competes with HPV for binding to Annexin A2 (AnxA2), pivotal for HPV cell entry. Here we investigate the mechanistic aspects of our hypothesis using transfection assays.

**Methods:**

HaCaT and HeLa cell lines were used to investigate the effects of shRNA transfection and nicotine exposure on HPV16-PsV-uptake. Cells were treated with Lipofectamine™ RNAiMAX for 48 or 72 hours with specific shRNA-concentrations, while nicotine was added to the cell medium at the indicated concentrations. Protein isolation, SLPI- and AnxA2-quantification, LDH cytotoxicity assessment, HPV16-PsV-uptake measurement, mRNA-isolation, cDNA-synthesis and RT-qPCR were performed.

**Results:**

*In vitro* transfection experiments with HPV16 pseudovirions (PsVs) showed that PsVs entered cells significantly better when SLPI was downregulated and significantly less when AnxA2 was downregulated. Nicotine exposure increased SLPI levels and reduced PsV uptake.

**Conclusions:**

The overexpression of SLPI caused by tobacco-smoking can hinder HPV cell entry by binding to AnxA2 and thus prevent successful HPV infection. Conversely, non-smokers have lower SLPI-levels, associated with an excess of unbound AnxA2, favoring HPV cell-entry. These findings support our hypothesis, suggesting a paradigm shift in understanding virus-related pathogenesis, particularly in the head and neck region, and the nature of HPV infection.

## Introduction

Head and neck squamous cell carcinoma (HNSCC) presents a significant public health concern, caused by both environmental exposure and viral infections. Among these factors, Tobacco consumption has been consistently linked to elevated risk of HNSCC, particularly affecting the larynx and hypopharynx (1, 2). Human papillomavirus (HPV) infections, predominantly HPV16, have emerged as significant contributors to tonsillar squamous cell carcinoma (TSCC), especially in non-smokers (3). Approximately 60% (Western Europe and the USA) to 90% (Scandinavia) of tonsillar squamous cell carcinomas are caused by HPV, whereas non-tonsillar cancers exhibit HPV positivity in only 10-30% of cases (3). Notably, the phenomenon of non-smokers predominantly developing HPV-positive cancers and smokers developing HPV-negative cancers remains unclear.

Previous own epidemiological studies [summarized in (4, 5)], encompassing biomaterials of over 1,100 patients, unveiled intriguing correlations between smoking habits, expression levels of the human secretory leukocyte protease inhibitor (SLPI), Annexin A2 (AnxA2) (for details see below*)*, and HPV infections in various head and neck and vulvar cancers, and also in non-neoplastic tonsillar tissue, leading to the following hypothesis: Smoking results in both increased SLPI and AnxA2 expression with higher SLPI than AnxA2 expression. When comparing AnxA2 versus SLPI expression, this relationship was nearly even in smokers, but non-smokers showed a continuous AnxA2-surplus. Our hypothesis postulates that tobacco smoking-driven SLPI overexpression leads to increased SLPI binding to AnxA2, thereby preventing HPV from binding to AnxA2, a crucial step for HPV cell entry. This mechanism provides a comprehensive explanation for the prevalence of HPV-negative carcinomas in smokers, in contrast to the higher likelihood of HPV-positive carcinomas in non-smokers, attributed to an abundance of unbound AnxA2, which can then be utilized by HPVs to enter and infect cells (4, 5).

### The secretory leukocyte protease inhibitor

Secretory leukocyte protease inhibitor (SLPI) was originally identified as a serine protease inhibitor found in the vicinity of pathogen entry (6). SLPI is an evolutionarily conserved pleiotropic protein expressed at mucosal surfaces, mainly by epithelial cells, including salivary glands, epidermis of the skin, and epithelia lining the respiratory, gastrointestinal, and genitourinary tract (7). It is also expressed by cells of the immune system (8–10), and exerts antimicrobial activity. SLPI maintains homeostasis in barrier tissues by preventing tissue destruction and regulating the threshold of inflammatory immune responses while protecting the host from infection [ (7), and references therein]. In the context of cancer, the role of SLPI was found to be contradictory. As summarized by Nugteren and Samsom (7), SLPI is described in the context of prevention and promotion of tumor growth or metastasis, even within the same tumor entity. These controversies can also be found in HNSCC: By some authors, high SLPI levels in HNSCC were found to have negative effects on invasive tumor growth and metastasis formation (11–13), accompanied by low SLPI levels in metastasized head and neck carcinomas (13). However, Takamura et al. (14) found that high SLPI promotes migration, invasion, and proliferation of oral squamous cell carcinomas. SLPI is not only involved in HPV infection but also in other viral infections of cells (15–18). SLPI was initially shown to inhibit HIV-1 infection of macrophages through direct interaction with host cell molecules, but not with the virus itself (15, 19). Later, Ma et al. (20) demonstrated that AnxA2 acts as a cellular cofactor that facilitates HIV-1 infection in macrophages and can be blocked by SLPI. Finally, Woodham et al. (21) revealed a pivotal finding: Not only does SLPI bind to AnxA2, but HPV can also bind to AnxA2, thereby playing a crucial role in HPV cell entry and infection.

### Annexin A2

AnxA2 can be found in the cytoplasm as a monomer and is expressed on the cell surface of basal epithelial cells as a heterotetramer (A2t) consisting of two AnxA2 monomers and a S100A10 dimer (22–24). AnxA2 plays a central role in cell adhesion, endocytosis, and exocytosis (25). AnxA2 is a crucial player in HIV-1 infection, directly interacting with HIV-1 proteins and impacting virus maturation; intriguingly, this process however is inhibited in the presence of SLPI (26). AnxA2 is also a receptor for respiratory syncytial virus infections (27). Similarly, the involvement of AnxA2 extends to HPV, where it also appears as an indispensable receptor in HPV infection, influencing viral attachment, internalization, and intracellular trafficking (21).

Investigating the association between SLPI and AnxA2 expression, smoking behavior, and HPV infection in HNSCC, we initially showed in *ex vivo* experiments that exposure to nicotine, a major component of tobacco, leads to an increase in SLPI expression in the human nasal mucosa, revealing the possible mechanisms underlying the consistent observation that smokers develop mostly HPV-negative HNSCC, whereas non-smokers develop HPV-positive HNSCC (28). In the present study, we conducted infection assays in HaCaT and HeLa cells using HPV16 pseudovirions (PsVs) to further validate the aforementioned mechanistic correlations. The rationale behind these experiments was to elucidate whether nicotine exposure influences SLPI and AnxA2 expression, subsequently impacting the successful entry of HPV16 PsVs into cells. We measured SLPI and AnxA2 gene and protein expression by RT-PCR and ELISA, respectively, and quantified the number of HPV16 PsVs successfully entering the cells through a Gaussia Glow Luciferase assay.

Thus, this study aimed to bridge the gap between epidemiological observations and cellular mechanisms and provide a deeper understanding of how tobacco exposure (specifically nicotine) might modulate the expression of SLPI and AnxA2, thus modulating HPV infections in human mucosa. The results of this study provide new insights into the complex interplay between environmental factors, viral infections, and cellular responses. Understanding these relationships may provide a basis for developing targeted preventive and therapeutic measures to combat HPV-related cancer.

## Methods

### Pseudovirion preparation

Pseudovirions were prepared as previously described (29, 30) with some modifications: 293TT cells were co-transfected with plasmids encoding humanized HPV L1 and L2 genes and a plasmid encoding the reporter enzyme Gaussia luciferase. For one 15cm dish, co-transfection with 17.5µg DNA was performed on 7 × 10^6^ 293TT cells using Lipofectamine according to the manufacturer’s instructions. Briefly, 17.5µg DNA were mixed with 70µl of reagent P3000 and 500µl OptiMEM. 54.3µl of TF_Lipo3000 were mixed with 500µl OptiMEM in another tube. Afterwards, the 2 tubes were mixed together and incubated for 10-15min. The transfection-mixture was added dropwise to 293TT cells, which were subsequently incubated at 37°C, 5% CO_2_ for 72hr. Absence of cell-culture contaminations was confirmed by the Multiplex cell Contamination Test.

For pseudovirion extraction, 293TT cells were harvested, and the cell pellet was resuspended in lysis buffer using one µl per mg pellet. The lysis buffer contains for 1ml: 1ml DPBS (+Mg+Ca); 58.3µl 10% Brij^®^ 58 and 6.7µl RNAse A/T cocktail. Cells were rotated for two days at 37°C to induce pseudovirion maturation. On day three, 250U of benzonase (Merck) were added after salt extraction. Treatment was performed for 1-hr incubation at 37°C. The pseudovirions were purified by Optiprep (Sigma) gradient, and fractions were stored at −80°C.

Specificity of pseudovirions was analyzed by infecting HeLa-cells in presence or absence of neutralizing antibodies: 50µl of iodixanol fractions F6 and F7 of pseudovirion preparations diluted 1:500 were added to wells of a 96 well plate followed by addition of 50µl of either guinea pig anti Gardasil 9 serum (GP_G9) at a dilution of 1:250 or the neutralizing monoclonal antibody 1.3.5.15 (1:1000). After incubation for 15min at RT, 50µl 5x194 HeLa-T-cells were added per well. All dilutions were performed in DMEM containing 10% FCS. Plates were incubated at 37°C in a humidified cell culture incubator for two days. To determine luciferase activity, 10µl of the supernatant were added to 100µl Gaussia Glow Juice (102542, p.j.k, Kleinbittersdorf, Germany) according to the manufacturer’s instructions. Relative light units (RLU) were determined in a Perkin Elmer Victor Nivo for 200ms 15min after addition of substrate. The results are shown in **Supplementary Figure 1**.

### Cell cultures, culture conditions, shRNA transfection- and nicotine exposure experiments

HaCaT and HeLa cells were used in this study, as they are two common human cell-lines used in HPV entry and life cycle research [ (21) and references therein]. HaCaT-cells (provided by O.J. Müller; original supplier: ATCC) are a spontaneously transformed aneuploid immortal keratinocyte cell-line derived from adult human skin (31). HeLa-cells (also provided by O. J. Müller; original supplier: ATCC) are human epithelial cells derived from cervical cancer. Both cell-lines were maintained in DMEM High Glucose (Capricorn, Ebsdorfergrund, Germany) supplemented with 10% FBS (Capricorn, Ebsdorfergrund, Germany), PenStrep (1%), and L-Glutamine (1%) (both: Gibco; Thermo Fisher, Darmstadt, Germany) at 37°C and 5% CO_2_.

HaCaT-cells were seeded at 100,000 cells/well and HeLa-cells at 60,000 cells/well into 12 well plates and incubated overnight at 37°C. The next day, cells for the shRNA experiments were incubated with 25nM or 50nM/well of the respective shRNA initially for 48 or 72h. shRNAs were purchased from Dharmacon (Horizon, Cambridge, United Kingdom): L-010741-00-0050, ON-TARGETplus Human ANXA2 (302) siRNA – SMARTpool with the following target sequences: CGACGAGGACUCUCUCAUU, AUCCAAGUGUCGCUAUUUA, AAAACCAGCUUGCGAAUAA, and GGAAGAAAGCUCUGGGACU. L-011391-00-0050, ON-TARGETplus Human SLPI (6590) siRNA – SMARTpool with the following target sequences: AGUCUGUCCUCCUAAGAAA, GGCCAAUGUUUGAUGCUUA, UGUGAAAGCUUGAUUCCUG, and UGACACUUGUGGCAUCAAA. D-001810-10-50, ON-TARGETplus Non-targeting Pool with the following target sequences: UGGUUUACAUGUCGACUAA, UGGUUUACAUGUUGUGUGA, UGGUUUACAUGUUUUCUGA, and UGGUUUACAUGUUUUCCUA.

shRNA-transfection of the cells was performed using the Lipofectamine™ RNAiMAX Transfection Reagent (Thermo Fisher, Darmstadt, Germany), following the manufacturer’s protocol for Forward Transfection of HeLa and HaCaT-cells, respectively.

In all experiments, where not only the effect of the shRNA alone, but also the effect of shRNA-incubation (SLPI- or AnxA2-knockdown) on HPV16 PsV-uptake was determined; shRNA-incubation was performed for 48h with 25nM shRNA followed by a media exchange with medium containing PsVs (provided by M. Müller; Research Program Infection and Cancer, German Cancer Research Center, Im Neuenheimer Feld 242, D69120 Heidelberg, Germany) at a dilution of 1:1000. The cells were incubated for further 24h, control incubations were performed with no shRNA. All experiments were performed three times in triplicate.

**Figure 1:**
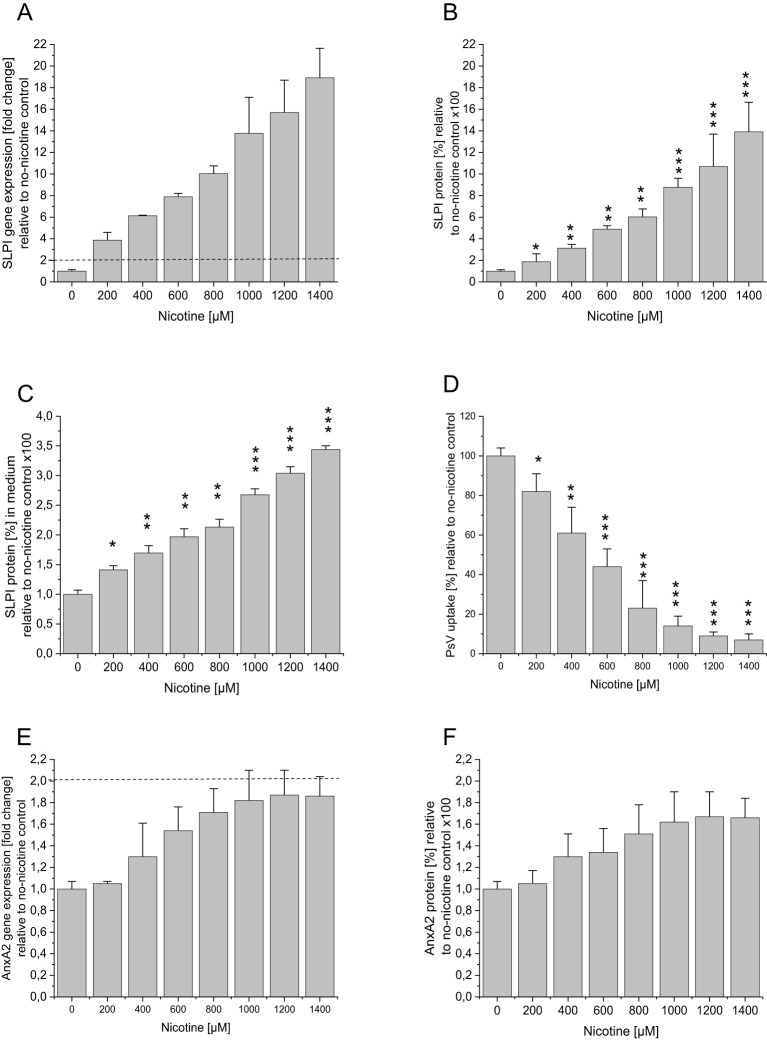
Effect of nicotine incubation on SLPI and AnxA2 gene and protein expression and HPV16 PsV uptake in HaCaT cells. HaCaT cells were seeded at 100,000 cells/well into 12 well plates and were incubated overnight at 37°C. After 24h medium containing nicotine at the indicated concentrations was added, after further 48h medium was replaced with nicotine and PsVs at a dilution of 1:1000. The control incubations were performed in the absence of nicotine. **(A)** SLPI gene expression as fold-change relative to no nicotine is shown, with the dotted line indicating significant changes >2 fold in relation to the no-nicotine control. In **(B)** SLPI protein expression in % relative to the no-nicotine control is shown. In addition, the SLPI levels in the cell supernatant were measured, and the results are shown in **(C)**. **(D)** shows the effect of nicotine treatment on PsV uptake. **(E)** AnxA2 gene expression as fold-change relative to no nicotine is shown, with the dotted line indicating significant changes >2 fold in relation to the no-nicotine control. In **(F)** AnxA2 protein expression in % relative to the no-nicotine control is shown. All panels show representative examples of three experiments all performed in triplicate, representing mean ± SD; * indicates p<0.05, ** p<0.01, and *** p<0.001, in relation to the no shRNA control.

To study the effect of nicotine, cells were seeded as described above. After 24h medium containing nicotine (Sigma Aldrich, Merck, Darmstadt, Germany) at the indicated concentrations (see **Figure 1**) was added. After further 48h medium was replaced with new medium containing nicotine, as before, and PsVs (provided by M. Müller) were added at a dilution of 1:1000. The control incubations were performed in the absence of nicotine. All experiments were performed three times in triplicate.

### Protein isolation

Cells were washed 2x with 1ml PBS without Ca/Mg (Gibco; Thermo Fisher, Darmstadt, Germany) and thereafter lysed with 250µl RIPA buffer (Thermo Fisher, Darmstadt, Germany), followed by three freeze-thaw cycles as follows: 30min liquid N_2_, followed by incubation for 5min at 30°C, 1000rpm, Thermomixer, Eppendorf (Eppendorf, Hamburg, Germany), followed by a final centrifugation at 4°C, 18000g for 20min (Eppendorf centrifuge 5430R, Eppendorf, Hamburg, Germany). The supernatant was transferred into a new reaction tube and stored at -80°C.

### SLPI- and AnxA2- protein determination

SLPI-protein levels were determined using the Human SLPI ELISA kit (RDR-SLPI-Hu-96T; Hycultec; Beutelsbach, Germany) according to the manufacturer’s protocol. AnxA2-protein levels were determined using the Human Anx2 ELISA Kit (ab264612; abcam, Rozenburg, The Netherlands) according to the manufacturer’s protocol. All samples were measured in triplicate. Instrument: Thermo Scientific Multiskan Sky (Thermo Fisher, Darmstadt, Germany).

### LDH cytotoxicity

To evaluate the possible toxic effects of nicotine exposure on HaCaT- and HeLa-cells, aliquots of the cell supernatants were collected at the end of the experiments and stored at -80°C until analysis. To measure cytotoxicity, the LDH Cytotoxicity Assay Kit (BAS-C2LD-100; Biozol, Diagnostica, Eching, Germany) was used according to the manufacturer’s protocol. All samples were measured in triplicate. Instrument: Thermo Scientific Multiskan Sky. (Thermo Fisher, Darmstadt, Germany).

### HPV16 PsV uptake

To study the effect of shRNA- or nicotine-incubation on HPV16 PsV-uptake, 10µl of cell culture supernatant was analysed using the Gaussia Glow-Juice Luciferase Assay kit (102542, p.j.k, Kleinbittersdorf, Germany) according to the manufacturer’s protocol. Controls without PsV were initially used to ensure that neither shRNAs nor nicotine had any influence on the luciferase activity of the medium. All samples were measured in triplicate. Instrument: Agilent - BioTek Citation5 imaging system and Gen5 software (Agilent Germany, Waldbronn, Germany).

### mRNA isolation, cDNA synthesis and RTqPCR

Cells were washed 2x with 1ml PBS without Ca/Mg (Gibco; Thermo Fisher, Darmstadt, Germany), lysed with 1ml peqGold TriFast, VWR (Darmstadt, Germany), and mRNA was isolated according to the manufacturer’s protocol. The resulting RNA-pellet was dissolved in 20µl DEPC water (Ambion, Thermo Fisher, Darmstadt, Germany) and stored at -80°C. RNA (200 ng) was transcribed into cDNA (cDNA synthesis kit AmpTec, Hamburg, Germany), according to the manufacturer’s protocol. RTqPCR and primer design were performed as described previously (32) using the following primers at an annealing temperature of 60°C: SLPI, Forward: 3’-AATgCCTggATCCTgTTgAC-5’; SLPI, Reverse: 3’-AAAggACCTggACCACACAg-5’; AnxA2, Forward: 3’-AACCgACgAggACTCTCTCA-5’; AnxA2, Reverse: 3’-CgCTgATCCACTTgggAACAT-5’. Primers for the house keeping gene β-actin were purchased from Promolgene (Berlin, Germany) and were used according to the manufacturer’s protocol. All samples were measured in duplicate using a Corbet Rotorgene (LTF, Wasserburg, Germany).

### Statistical analysis

RT−qPCR data analysis was performed according to the ΔΔCt method (33), using the Ct-value of the housekeeping gene β-actin for normalization. This equation was used to determine the effect of shRNA incubation on the expression of the target genes (SLPI and AnxA2). Expression levels were normalized against those obtained in no shRNA controls, and these values were set to 100%. To calculate the effect of nicotine-exposure fold changes of the expression levels of the normalized target genes (SLPI and AnxA2), the following equations were used: 1/2^(ΔCt no nicotine-ΔCt various nicotine concentrations for increases in nicotine−related gene expression. With no-nicotine values set as “1”. Protein levels were calculated using the HycultBiotech ELISA assay results software (https://www.assayfit.com/company/hycult/hycult-input-new.html) with no shRNA controls and no nicotine controls set as 100%. Fold change levels ≥2 obtained by RT-qPCR analysis were considered significantly different (35). Statistical differences between protein levels and PsV uptake were calculated by one-way ANOVA followed by Dunnett post-test, where appropriate. All tests were performed using SPSS 20.0, and statistical significance was set at p ≤0.05.

## Results

### Role of SLPI and AnxA2 in the uptake of HPV16 PsV

**Figure 2:**
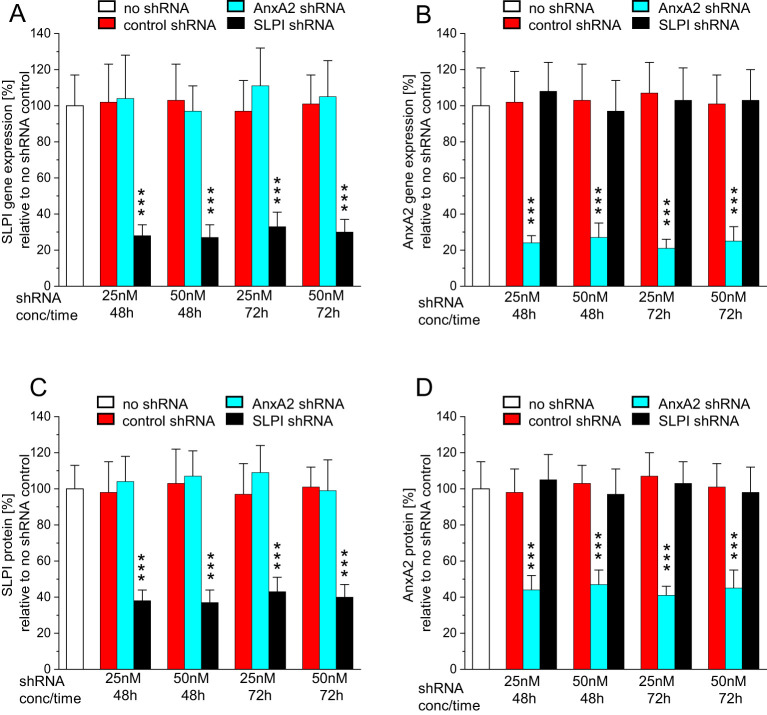
Incubation with AnxA2 and SLPI shRNA results in successful down regulation of the respective mRNAs and proteins in HaCaT cells. HaCaT cells were seeded at 100,000 cells/well into 12 well plates and were incubated overnight at 37°C. After 24h cells were incubated with 25nM or 50nM/well of control, AnxA2, or SLPI shRNA for 48 or 72h. All panels show representative examples of three experiments performed in triplicate, representing the mean ± SD, with *** indicating p<0.001 in relation to the no shRNA control. **(A)** SLPI gene expression as fold-change relative to no shRNA is shown. In **(B)** AnxA2 gene expression as fold change relative to no shRNA is shown, and **(C, D)** show SLPI and AnxA2 protein expression in % relative to the no shRNA control. In all panels, a significant downregulation of the target mRNA and protein with no cross-reactivity was observed.

To confirm the previously demonstrated role of SLPI and AnxA2 in HPV16 PsV cell-entry (23, 36), we initially showed that incubation of HaCaT- and HeLa-cells with 25nM and 50nM shRNA for 48h or 72h resulted in the downregulation of SLPI and AnxA2 gene and protein expression, respectively, with no significant cross-reactivity (**Figures 2A–D**; the Results obtained in HeLa-cells are shown in **Supplementary Figure 2**). Since the 72h incubation did not yield further downregulation of gene- or protein-levels, all subsequent experiments involving additional HPV16 PsV-incubation were carried out after 48h of shRNA incubation.

**Figure 3:**
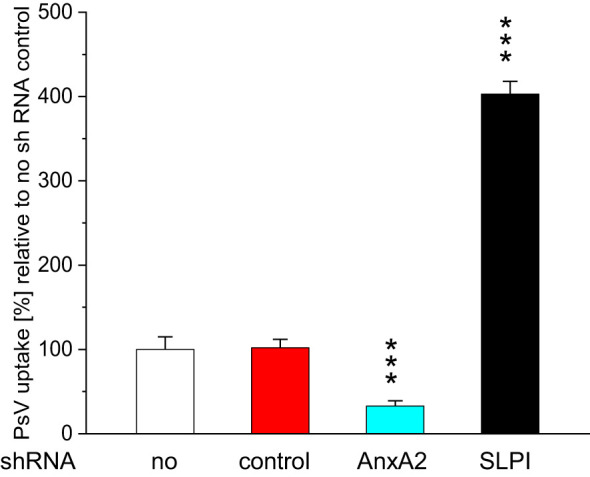
Effect of incubation with AnxA2 and SLPI shRNA on HPV16 PsV uptake in HaCaT cells. HaCaT cells were seeded at 100,000 cells/well into 12 well plates and were incubated overnight at 37°C. After 24h cells were incubated for 48h with either 25nM control shRNA (red bars) and 25nM AnxA2 or SLPI shRNA, turquoise and black bars, respectively. After 48h new medium (1 ml) containing PsVs at a dilution of 1:1000 was added, and the cells were incubated for further 24h, control incubations (white bars) were carried out for further 24h with medium without PsVs. Incubating cells with AnxA2 shRNA significantly decreased PsV uptake, whereas incubation with SLPI shRNA significantly increased PsV uptake. The image shows a representative example of three experiments all performed in triplicate, representing the mean ± SD, with *** indicating p<0.001, in relation to the no shRNA control.

The observed downregulation of AnxA2 and SLPI significantly reduced HPV16 PsV uptake in the case of AnxA2-downregulation and significantly increased HPV16 PsV uptake in the case of SLPI-downregulation (**Figure 3**). Similar, though slightly less pronounced, results were obtained when incubating HeLa-cells (**Supplementary Figure 3**).

### Nicotine hinders SLPI and AnxA2-mediated uptake of HPV16 PsV

To study the effect of nicotine on SLPI and AnnxA2, we incubated HaCaT- and HeLa-cells with various concentrations of nicotine. **Figure 1** illustrates the results of the nicotine-incubation in HaCaT-cells. In agreement with our patient-derived data, nicotine-incubation led to a significant dose-dependent increase in SLPI gene- and protein expression (**Figures 1A, B**). AnxA2-levels, however, showed a slight, albeit non-significant, increase (<2-fold increase in gene expression and p>0.05 for protein expression; **Figures 1E, F**). Similar, but slightly less pronounced, results were obtained when incubating HeLa-cells (**Supplementary Figure 4**). Additionally, in HeLa-cells, nicotine induced an increase in SLPI-expression only from 600µM onwards (gene and protein). To investigate whether higher nicotine concentrations might lead to a further increase in SLPI-expression a second round of experiments with HeLa-cells and higher nicotine concentrations was performed, with, however, no further increase in SLPI-expression levels (gene and protein; **Supplementary Figures 5A, B**). To further confirm the role of SLPI as an agent competing with HPV for cell entry, SLPI-protein was measured in the cell supernatant, showing that increasing nicotine concentrations led to increased SLPI-protein levels in the cell supernatant (results for HaCaT-cells are shown in **Figure 1C** and for HeLa-cells in **Supplementary Figures 4C, 5C**). Next, we used the cell supernatants of the nicotine experiments to determine the possible effects of nicotine and the observed nicotine-related increase in SLPI-protein on HPV16 PsV-uptake, by performing Gaussia-Glow measurements. The results for HaCaT-cells are shown in **Figure 1D** (those for HeLa-cells are shown in the **Supplementary Figures 4D, 5D**). It can be seen that increasing nicotine levels, which are accompanied by increasing SLPI-levels (**Figures 1A–C**) result in decreased PsV-uptake. Similar, albeit less pronounced results were obtained in HeLa-cells (**Supplementary Figures 4, 5**).

To study the possible cytotoxic effect of nicotine LHD-assays were performed in HaCaT-and HeLa-cell supernatants, demonstrating that nicotine, at all concentrations tested, had no effect on cell viability (**Supplementary Figure 6**).

## Discussion

The often described but as yet not fully understood observation of HPV-negative carcinomas in smokers and HPV-positive carcinomas in non-smokers finds an intriguing explanation in the results of the study presented here and is supported by our previous epidemiological studies, involving biomaterials of more than 1,100 patients (4, 5). These patient-derived data showed a consistent correlation between the patients’ smoking habits and the expression levels of SLPI and AnxA2, as well as HPV infection status.

Based on our own epidemiologic patient data, we have already hypothesized a link between tobacco smoking and the upregulation of SLPI: Smoking leads to increased expression of SLPI and facilitates the binding of SLPI to AnxA2, preventing the successful binding of HPV to AnxA2 in a competitive environment - a crucial step for HPV entry into the cell, as previously shown (21, 34). The here presented data confirm that SLPI and AnxA2 are involved in HPV-uptake. We successfully downregulated SLPI and AnxA2 gene, and consequently protein expression using shRNA. The downregulation of AnxA2 resulted in a significant reduction in HPV16 PsV-uptake. Similarly, it was shown that increasing amounts of anti-AnxA2 antibodies led to reduced uptake of HPV16 PsV (21, 34) demonstrating together with our here presented results that AnxA2 plays a crucial role in HPV-cell entry and subsequently in HPV infection of the cells.

Furthermore, the two aforementioned studies (21, 34) showed that incubation of HaCaT and HeLa cells with increasing amounts of recombinant human SLPI resulted in decreased uptake of HPV16-PsVs, suggesting competition between SLPI and HPV16-PsVs for binding to AnxA2 and allowing cell entry of either SLPI or HPV. The results presented here support the aforementioned findings, but using a reverse approach: We downregulated the SLPI mRNA, and consequently the protein, and found a significant increase in the amount of HPV16-PsVs entering cells. The novelty of the results presented here is that our results show that direct downregulation (utilizing shRNA) and not indirect effects (incubation of the cells with recombinant proteins) lead to an altered HPV entry.

The novel and intriguing aspect of the present study was to investigate the role of nicotine in this context and in relation to HPV-associated HNSCC carcinogenesis. To this end, we incubated two different cell lines with nicotine to mimic the effects of tobacco smoking on SLPI and AnxA2 expression in cell culture models. We successfully demonstrated that incubation of HaCaT and HeLa cells with increasing amounts of nicotine led to significantly increased SLPI mRNA and protein expression, accompanied by significantly reduced HPV16PsV uptake, with AnxA2 expression not being significantly affected by nicotine. The notable reduction in the uptake of HPV16 PsVs, particularly pronounced in HaCaT cells when compared to HeLa cells, provides valuable insights into cell-specific responses to stimuli associated with smoking. HeLa cells are derived from HPV-positive cervical cancer, whereas HaCaT cells are derived from adult human skin, with cells spontaneously transformed and immortalized, which might explain the differences in test results. Nevertheless, these cell lines are frequently used in HPV entry and life cycle studies [21, 34 and references therein]. Altogether, the observations presented here align with the results from our patient-derived tissue samples, thereby emphasizing the consistency of our findings. In particular, these data for the first time demonstrate a mechanistic explanation why mostly smokers suffer from HPV-negative HNSCCs while non-smokers develop HPV-positive cancers. Our results can therefore also very well explain the exponentially increasing incidence of HPV-positive oropharyngeal carcinomas despite declining smoking rates in the United States (35, 36). We cannot exclude, though, the possibility that nicotine recruits other (co-)factors, resulting in increased SLPI levels; for example, tobacco has been linked to the alteration and induction of immune response, which might affect SLPI levels (37). However, it has been clearly shown that SLPI is a ligand of AnxA2, and increased SLPI levels lead to reduced HPV entry into mucosal cells (21, 34). Even if previously unknown cofactors are involved, the present study has shown that a nicotine-induced increase in SLPI mRNA and protein levels together with the observed reduced uptake of HPV16-PsVs is a strong indicator of a direct link between tobacco use, nicotine being a major component of tobacco smoke, and the occurrence of HPV-negative HNCSS. This statement is supported by the fact that we could also see in patients with tonsillar hyperplasia, a benign condition of the tonsils not regularly accompanied by inflammation-associated processes, a significant increase of SLPI in smoking patients when compared to non-smoking patients (38).

The data presented here illustrate the interplay of environmental factors, viral infections and cellular reactions in the example of HNSCC and beyond. This seems to provide a comprehensible and plausible answer to the question regarding the apparent high prevalence of HPV-positive HNSCC in non-smoking patients and vice versa.

Limitations of our experimental approach might be that i) we used HPV pseudovirions as surrogate for native HPV particles or HPV quasivirions. Differences between the former and the latter have been outlined previously (39) and the fact that ii) we only used HPV PsVs of one genotype, HPV16. HPV16 however is the most common HPV type found in HNSCC (40). Another limitation of the study might be that iii) we used only nicotine and not e.g. tobacco smoke condensate, however, in previous *ex vivo* studies utilizing nasal mucosal tissue we could demonstrate that nicotine plays a crucial role in the upregulation of SLPI. Future experiments should include tobacco smoke condensate to ensure that the entire array of compounds have the same effect as those seen here with using only nicotine.

Nonetheless, the results presented here, not only open up new avenues for understanding the molecular mechanisms underlying HPV-associated and non-HPV-associated HNSCC, which represents a paradigm shift in this field, but also fundamentally contributes to our understanding of the HPV infection mode. These results may serve as the basis for the development of targeted prevention and intervention strategies for HPV-associated malignancies.

## Data Availability

The original contributions presented in the study are included in the article/**Supplementary Material**. Further inquiries can be directed to the corresponding author.

## In memoriam

Dedicated to Harald zur Hausen.
