# Supplementary material for: Mechanistic insights into HPV-positivity in non-smokers and HPV-negativity in smokers with head and neck cancer
Source: Front Oncol. 2025 Jan 9;14:1484319. doi: 10.3389/fonc.2024.1484319 (PMC11754403; doi:10.3389/fonc.2024.1484319)
Supplement: Supplementary file 1 [file DataSheet1.docx]

Supplementary Figure 1


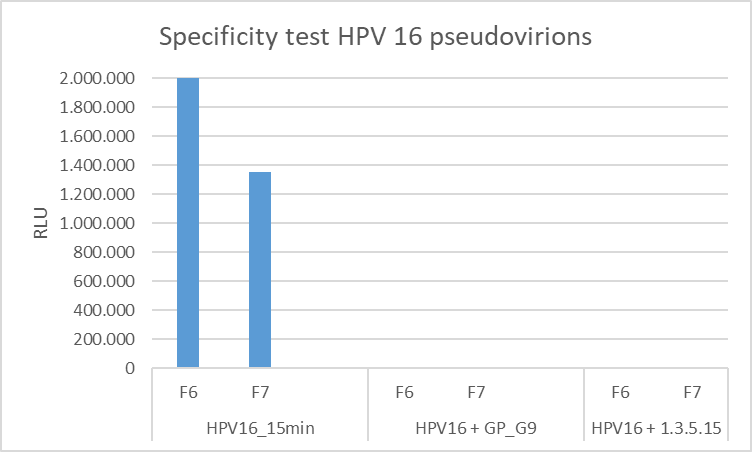


**Supplementary Figure 1: Specificity of HPV 16 pseudovirions.**

To verify specificity, Hela T cells were transduced with HPV 16 pseudovirions (fractions F6 and F7) encoding for Gaussia luciferase in presence or absence of neutralizing polyclonal antiserum GP_G9 or neutralizing monoclonal antibody 1.3.5.15. These fractions were used in the experiments presented here.
