# Supplementary material for: Mechanistic insights into HPV-positivity in non-smokers and HPV-negativity in smokers with head and neck cancer
Source: Front Oncol. 2025 Jan 9;14:1484319. doi: 10.3389/fonc.2024.1484319 (PMC11754403; doi:10.3389/fonc.2024.1484319)
Supplement: Supplementary file 2 [file DataSheet2.docx]

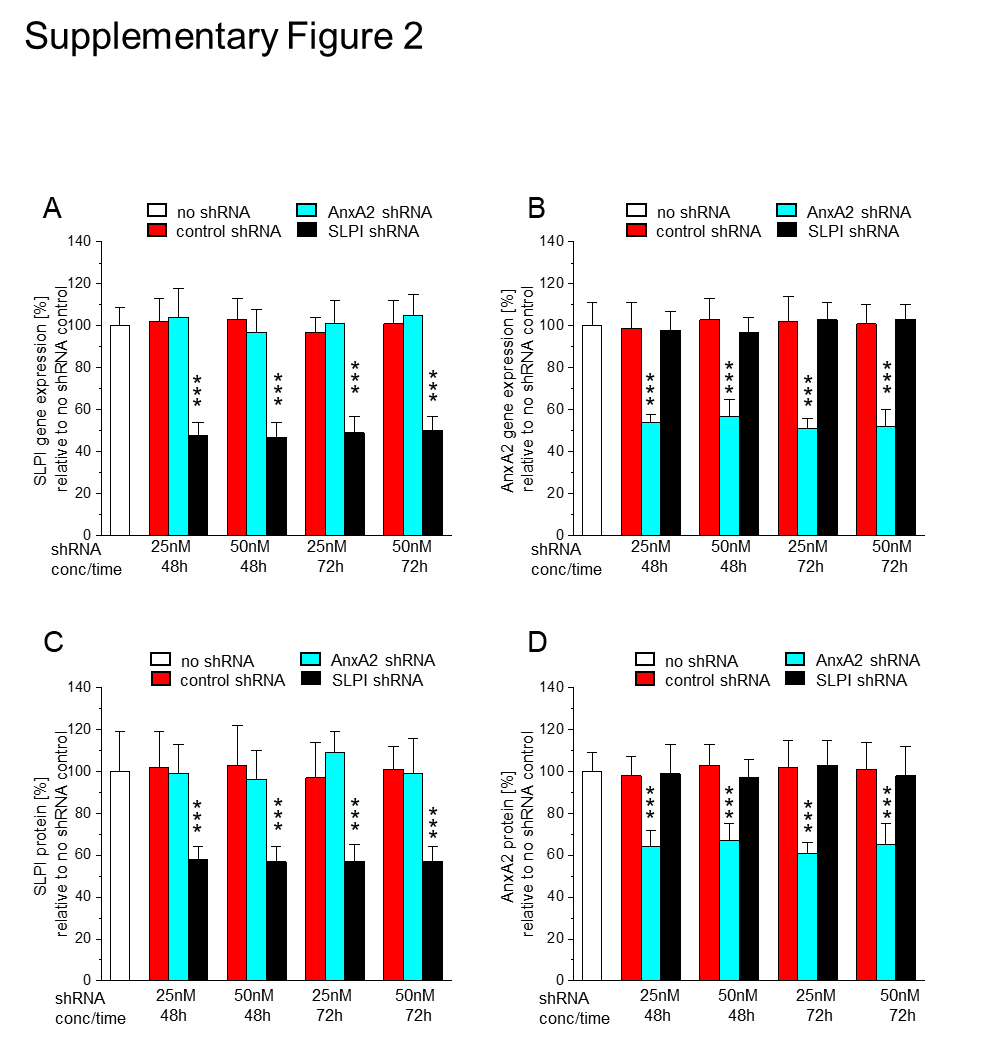


**Supplementary Figure 2. Incubation with AnxA2 and SLPI shRNA results in successful down regulation of the respective mRNAs and proteins.**

HeLa cells were seeded at 60.000 cells/well into 12 well plates and were incubated overnight at 37°C. After 24h cells were incubated with 25nM or 50nM/well of control, AnxA2, or SLPI shRNA for 48 or 72h. All panels show representative examples of three experiments performed in triplicate, representing the mean±SD, with *** indicating p<0.001 in relation to the no shRNA control. Supplementary Figure 2A: SLPI gene expression as fold-change relative to no shRNA is shown. In Supplementary Figure 2B, AnxA2 gene expression as fold change relative to no shRNA is shown, and Supplementary Figure 2C and 2D show SLPI and AnxA2 protein expression in % relative to the no shRNA control. In all panels, a significant downregulation of the target mRNA and protein with no cross-reactivity was observed.
