# Supplementary material for: Mechanistic insights into HPV-positivity in non-smokers and HPV-negativity in smokers with head and neck cancer
Source: Front Oncol. 2025 Jan 9;14:1484319. doi: 10.3389/fonc.2024.1484319 (PMC11754403; doi:10.3389/fonc.2024.1484319)
Supplement: Supplementary file 3 [file DataSheet3.docx]

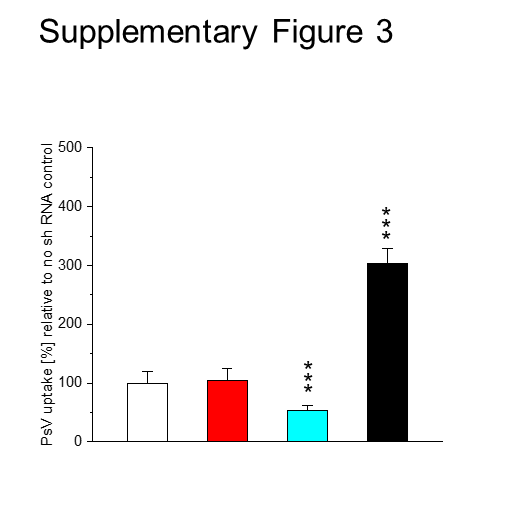


**Supplementary Figure 3. Effect of incubation with AnxA2 and SLPI shRNA on HPV16 PsV uptake in Hela cells**

HeLa cells were seeded at 60,000 cells/well into 12 well plates and were incubated overnight at 37°C. After 24h cells were incubated for 48h with either 25nM control shRNA (red bars) and 25nM AnxA2 or SLPI shRNA, turquoise and black bars, respectively. After 48h new medium (1 ml) containing PsVs at a dilution of 1:1000 was added, and the cells were incubated for further 24h, control incubations (white bars) were carried out for further 24h with medium without PsVs. Incubating cells with AnxA2 shRNA significantly decreased PsV uptake, whereas incubation with SLPI shRNA significantly increased PsV uptake. Supplementary Figure 3 shows a representative example of three experiments all performed in triplicate, representing the mean±SD, with *** indicating p<0.001, in relation to the no shRNA control.
