# Supplementary material for: Mechanistic insights into HPV-positivity in non-smokers and HPV-negativity in smokers with head and neck cancer
Source: Front Oncol. 2025 Jan 9;14:1484319. doi: 10.3389/fonc.2024.1484319 (PMC11754403; doi:10.3389/fonc.2024.1484319)
Supplement: Supplementary file 5 [file DataSheet5.docx]

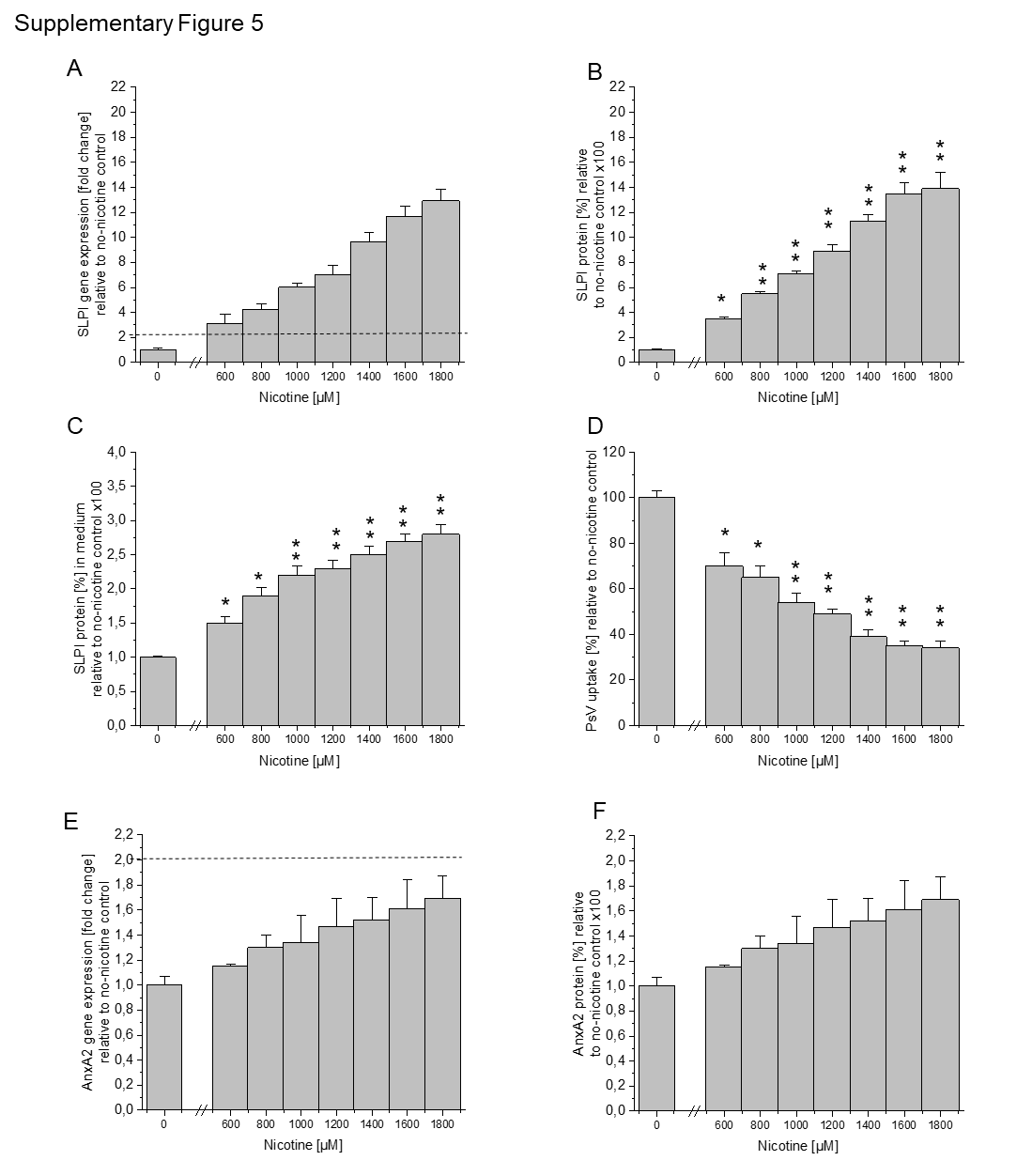


**Supplementary Figure 5. Effect of nicotine incubation on SLPI and AnxA2 gene and protein expression and HPV16 PsV uptake in HeLa cells**

HeLa cells were seeded at 60,000 cells/well into 12 well plates and were incubated overnight at 37°C. After 24h medium containing nicotine at the indicated concentrations was added, after further 48h medium was replaced with nicotine and PsVs at a dilution of 1:1000. The control incubations were performed in the absence of nicotine. Figure 5A: SLPI gene expression as fold-change relative to no nicotine is shown. In Figure 5B SLPI protein expression in % relative to the no-nicotine control is shown. In addition, the SLPI levels in the cell culture medium were measured, and the results are shown in Figure 5C. Figure 5D shows the effect of nicotine treatment on PsV uptake. Figure 5E shown AnxA2 gene expression as fold-change relative to no nicotine is shown. In Figure 5F AnxA2 protein expression in % relative to the no-nicotine control is shown. All panels show representative examples of three experiments all performed in triplicate, representing mean±SD; * indicates p<0.05, ** p<0.01, and *** p<0.001, in relation to the no shRNA control. The dotted lines indicate significant changes >2 fold of gene expression in relation to the no-nicotine control.
