# Supplementary material for: Mechanistic insights into HPV-positivity in non-smokers and HPV-negativity in smokers with head and neck cancer
Source: Front Oncol. 2025 Jan 9;14:1484319. doi: 10.3389/fonc.2024.1484319 (PMC11754403; doi:10.3389/fonc.2024.1484319)
Supplement: Supplementary file 6 [file DataSheet6.docx]

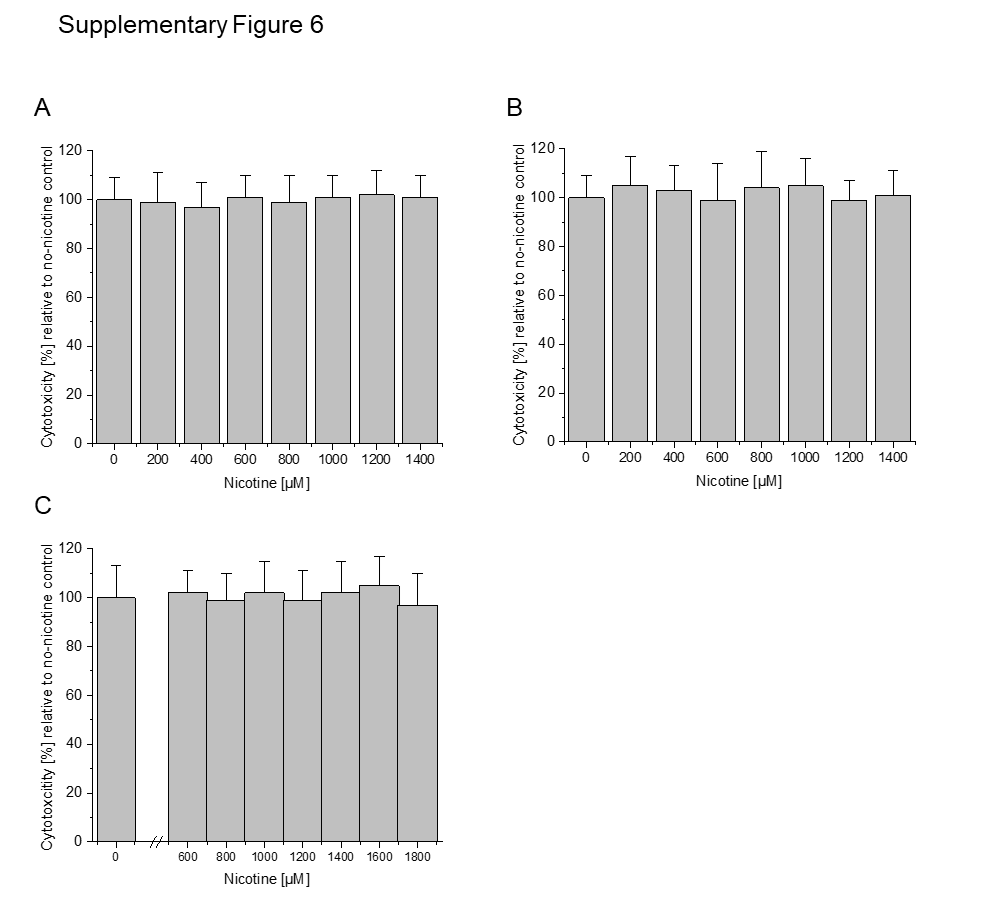


**Supplementary Figure 6. Effect of nicotine incubation on cell viability of HaCat and HeLa cells**

Cell culture supernatants were tested in an LDH assay. Showing in Figure A the results obtained in HaCat cells and in Figure B and C the results obtained in HeLa cells. All panels show Cytotoxicity in % relative to the no-nicotine control and are representative examples of three experiments all performed in triplicate, depicting mean±SD.
